# Ashtrays and Signage as Determinants of a Smoke-Free Legislation’s Success

**DOI:** 10.1371/journal.pone.0072945

**Published:** 2013-09-04

**Authors:** Constantine I. Vardavas, Israel Agaku, Evridiki Patelarou, Nektarios Anagnostopoulos, Chrysanthi Nakou, Vassiliki Dramba, Gianna Giourgouli, Paraskevi Argyropoulou, Antonis Antoniadis, Konstantinos Gourgoulianis, Despoina Ourda, Lambros Lazuras, Monique Bertic, Christos Lionis, Gregory Connolly, Panagiotis Behrakis, on behalf of the Hellenic Air Monitoring Study Investigators

**Affiliations:** 1 Center for Global Tobacco Control, Department of Social and Behavioral Sciences, Harvard School of Public Health, Boston, Massachusetts, United States of America; 2 Smoking and Lung Cancer Research Center, Hellenic Cancer Society, Athens, Greece; 3 Clinic of Social and Family Medicine, Department of Social Medicine, University of Crete, Heraklion, Greece; 4 Department of Medicine, Aristotelio University of Thessaloniki, Thessaloniki, Greece; 5 Department of Pulmonary Medicine, General Hospital of Serres, Serres, Greece; 6 Department of Respiratory Medicine, University of Thessaly, Thessaly, Greece; 7 South Eastern European Research Center, Thessaloniki, Greece; 8 Biomedical Research Foundation of the Academy of Athens, Athens, Greece; 9 Department of Environmental Health, Harvard School of Public Health, Boston, Massachusetts, United States of America; University of Montana, United States of America

## Abstract

**Introduction:**

Successful smoke-free legislation is dependent on political will, enforcement and societal support. We report the success and pitfalls of a non-enforced nationwide smoke-free legislation in Greece, as well as ways in which compliance and enforcement-related factors, including ashtrays and signage, may impact indoor secondhand smoke (SHS) concentrations.

**Methods:**

A follow-up study of venues (n = 150, at baseline, n = 75 at 2-year follow-up) in Greece assessed indoor particulate matter with a diameter less than 2.5 micrometers (PM*_2.5_*) concentrations attributable to SHS smoke every six months for two years (n = 455 venue/measurements).

**Results:**

Following the implementation of the 2010 smoke-free legislation, mean PM_2.5_ concentrations attributable to SHS fell from 175.3 µg/m^3^ pre-ban to 84.52 µg/m^3^ immediately post-ban, increasing over subsequent waves (103.8 µg/m^3^ and 158.2 µg/m^3^ respectively). Controlling for potential influential factors such as ventilation, time of day, day of week, city and venue type, all post-ban measurements were still lower than during the pre-ban period (Wave 2 beta: −118.7, Wave 3 beta: −87.6, and Wave 4 beta: −69.9). Outdoor or indoor signage banning smoking was not found to affect SHS concentrations (beta: −10.9, p = 0.667 and beta: −18.1, p = 0.464 respectively). However, ashtray or ashtray equivalents were strong determinants of the existence of indoor SHS (beta: +67 µg/m^3^, p = 0.017).

**Conclusions:**

While the public may be supportive of smoke-free legislation, adherence may decline rapidly if enforcement is limited or nonexistent. Moreover, enforcement agencies should also focus on the comprehensive removal of ashtray equivalents that could act as cues for smoking within a venue.

## Introduction

Exposure to secondhand smoke (SHS) has been linked to disease, premature death and disability. Estimated to cause over 400,000 deaths due to ischemic heart disease and an additional 36,900 from asthma annually in the United States, SHS exposure is a pertinent public health issue [1]–[3]. In response to the plethora of evidence supporting the need to minimize exposure to SHS, a number of countries around the world have proceeded to adopt smoke-free legislation with clear health benefits. A 2010 US, Institute of Medicine report reviewed 11 key epidemiologic studies evaluating the incidence of acute coronary events before and after the introduction of smoke-free policies in public areas and workplaces. The results consistently showed decreases in the rate of acute cardiac death after the implementation of smoking bans [2]. Similarly, studies examining the immediate effects on the pulmonary system after the passage of smoke-free laws have indicated reductions in respiratory symptoms and hospitalization [4].

The global need to adopt smoke-free legislation is summarized in the directives of Article 8 of the Framework Convention on Tobacco Control (FCTC), which calls for the adoption of smoke-free legislation as a key component of tobacco control within ratifying countries [5]. However, while the FCTC provides guidance, the adoption and maintenance of smoke-free legislation is the responsibility of regional political leadership, enforcement agencies and society itself. With the above in mind, we aimed to evaluate the components associated with the adoption and maintenance of the 2010 Greek smoke-free legislation [6]–[7]. Given that enforcement was relatively inexistent after the first month, this framework allows us to examine a) the factors associated with the success and pitfalls of non-enforced nationwide smoke-free legislation and b) the ways in which factors of enforcement, such as ashtrays and signage, may impact indoor SHS concentrations.

## Methods

### Study Design

The Hellenic Air Monitoring Study (HAMS) is a national longitudinal cohort of hospitality venues within five regions of Greece, within which indoor air pollution attributable to SHS is assessed at six-month intervals (waves) within the cities of Athens, Heraklion, Serres, Larissa and Thessaloniki. These cities were selected to geographically sample Southern (Heraklion), Central (Athens, Larissa) and Northern (Serres, Thessaloniki) areas in Greece. A convenience sample of 150 venues (30 from each city) was selected at baseline based on accessibility and popularity. A comprehensive list of hospitality venues for each city was not available. Measurements began during the pre-ban period of March–May 2010 (Wave 1), and we followed up using the same venues 6 months later (Wave 2) immediately after the implementation of the smoking ban (October– December 2010). Wave 3 and Wave 4 follow-up were performed in March-May 2011 and October-December 2011 respectively. An overview of the venues, cities and time-points of indoor air sampling is provided in **Table 1** **.** In total, 445 venue exposure measurements were performed during this two-year period. Out of the 150 venues assessed at baseline, 19 venues were lost due to financial closure (Athens = 11, Crete = 6, Thessaloniki = 2), while 53 were lost due to the inability to assess venues within Larissa and Serres in Wave 4 (Larissa = 28, Serres = 25) due to the unfortunate sudden loss of a researcher.

**Table 1:** Characteristics of the Hellenic Air Monitoring Study by wave and site, Greece, 2010–2011.

	Wave 1	Wave 2	Wave 3	Wave 4
**Smoking ban status**	Partial Ban	Complete Ban	Complete Ban	Complete Ban
**Measurement months**	Apr–May 2010	Oct–Dec 2010	Apr–Jun 2011	Oct–Dec 2011
**Time from baseline (months)**	0	6	12	18
**Overall follow up rate %(n)**	100 (148)	98.7 (146)	50.7 (75)	51.4 (76)
**Venue type**				
* Café %(n)*	43.7 (62)	46.4 (65)	42.7 (32)	41.1 (30)
* Bar %(n)*	41.6 (59)	39.3 (55)	45.3 (34)	46.6 (34)
* Restaurant %(n)*	14.8 (21)	14.3 (20)	12.0 (9)	12.3 (9)
**City** 1				
* Thessaloniki %(n)*	20.3 (30)	21.2 (30)	–	36.8 (28)
* Serres %(n)*	19.6 (29)	20.6 (29)	–	5.3 (4)
* Larissa %(n)*	18.9 (28)	17.1 (25)	33.3 (25)	–
* Crete %(n)*	20.3 (30)	21.2 (30)	40.0 (30)	31.6 (24)
* Athens %(n)*	20.9 (31)	19.9 (29)	26.7 (20)	26.3 (20)

**Abbreviations**: n = number of sampled venues.

1 In total 19 venues were lost due to closure (Athens = 11, Crete = 6, Thessaloniki = 2), while 53 were lost due to the inability to assess those cities in Wave 4 (Larissa = 28, Serres = 25).

Further details regarding the HAMS and the initial follow-up have been previously published [7].

### Air Sampling Methodology

Measurements were performed with the use of a TSI Sidepak AM510, using standardized methodology for monitoring indoor air pollution attributable to SHS (Calibration factor 0.32, flow rate 1.7 L/min). This methodology has been used previously to assess indoor particulate matter concentrations, with a diameter less than 2.5 micrometers (PM*_2.5_*) attributable to SHS, in numerous studies [8]–[10]. Although SHS is not the only source of PM*_2.5_* concentrations as external air pollution, candles or cooking may also increase indoor PM*_2.5_* concentrations, they can be used as a proxy for SHS exposure. In the absence of other indoor sources, we subtracted outdoor concentrations from indoor concentrations to obtain the concentrations of PM*_2.5_* attributable to SHS [9]–[10]. The TSI Sidepak was set to record the data at 1-minute intervals averaging the previous 60-second measurements. During data cleaning, first and last 1-minute measurements were discarded, while all measurements lasted at least 30 minutes. We then averaged the minute-logged measurements noted between entering and exiting each venue. Outdoor levels were collected by assessing for 3 minutes the outdoor PM*_2.5_* concentrations, which we defined as being 50 meters from the venue and free of any source of SHS. The average outdoor PM*_2.5_* concentrations were 12 µg/m^3^, with a range between 5 µg/m^3^ and 25 µg/m^3^.

In order to ensure consistency between measurements, field researchers were trained together on a number of pilot venues that were simultaneously assessed for their descriptive characteristics and indoor PM_2.5_ concentrations. During data collection, sampling was discreet to avoid altering the employees’ and patrons’ normal behavior. Descriptive information collected from each venue included the number of cigarettes lit during the measurement, people in each venue, venue size and other factors that might affect the data (e.g., candles or cooking in the venue). Smoker density was calculated by dividing the average number of burning cigarettes in each venue by the estimated room volume and was expressed as burning cigarettes per 100 m^3^. Open windows, as well as doors or sliding walls, were also noted and were used throughout the year given the mild Mediterranean climate. According to the Hellenic National Meteorological Service (EMY), the average temperatures between waves were similar.

### Exposures and Outcomes

The primary outcome was the average PM*_2.5_* levels attributable to SHS within the venues. Other secondary outcomes were average smoker density and average number of burning cigarettes. All outcomes of interest were measured on a continuous scale. The predictors of interest included time of day (daytime: before 8 pm vs. nighttime after 8 pm); ventilation (open/closed based on the existence of open windows or sliding glass walls); day of week (we categorized weekend as Friday, Saturday and Sunday vs. weekday if otherwise); type of venue (cafes, bars, or restaurants based on the basic service provided, namely coffee, alcohol, or food respectively); and city (to investigate the role of regional enforcement on the indoor levels of SHS).

During Waves 3 and 4, data was collected on the existence of signage or other inter-venue characteristics used as proxies for the strength of implementation and management support of smoking regulation in the hospitality venues. These variables, were dichotomized as “Yes” or “No” and included pro-tobacco control initiatives, such as the presence of signage on the door banning smoking, signage indoors banning smoking, and signage indoors banning smoking on the tables and bars. Anti-tobacco control initiatives were also documented, including the presence of ashtrays on tables (i.e. decorative candle holders, vases, plastic cups) and signage on the door opposing the smoking ban (used by pro-smoking groups against the smoking ban).

### Statistical Analysis

To determine the significance of changes in PM*_2.5_* levels between pair-wise wave measurements, we used a paired *t*-test at the alpha = 5% level. Results are presented as means, standard errors and ranges. Descriptive graphs are also provided. For each of the signage variables, we conducted a Fisher’s exact test to determine the relationship with venue and city. Descriptive statistics are also provided for these variables, with proportions expressed in percentages.

Because data were collected longitudinally with sequential measurements over four waves, we accounted for correlation for within-wave measurements and heterogeneous variability in the measurements in order to obtain valid estimates. Variables were included into the multivariate model based on their significance on univariate analysis at a significance level of alpha = 0.1. All selected variables (wave, ventilation, time of day, day of week, city and venue) were significant at alpha = 0.1 and thus included into the final multivariate mixed effects linear regression model. All analyses were performed with SAS Statistical software V.9.

## Results

### Indoor Air PM_2.5_ Concentrations Attributable to SHS

**Figure 1:**
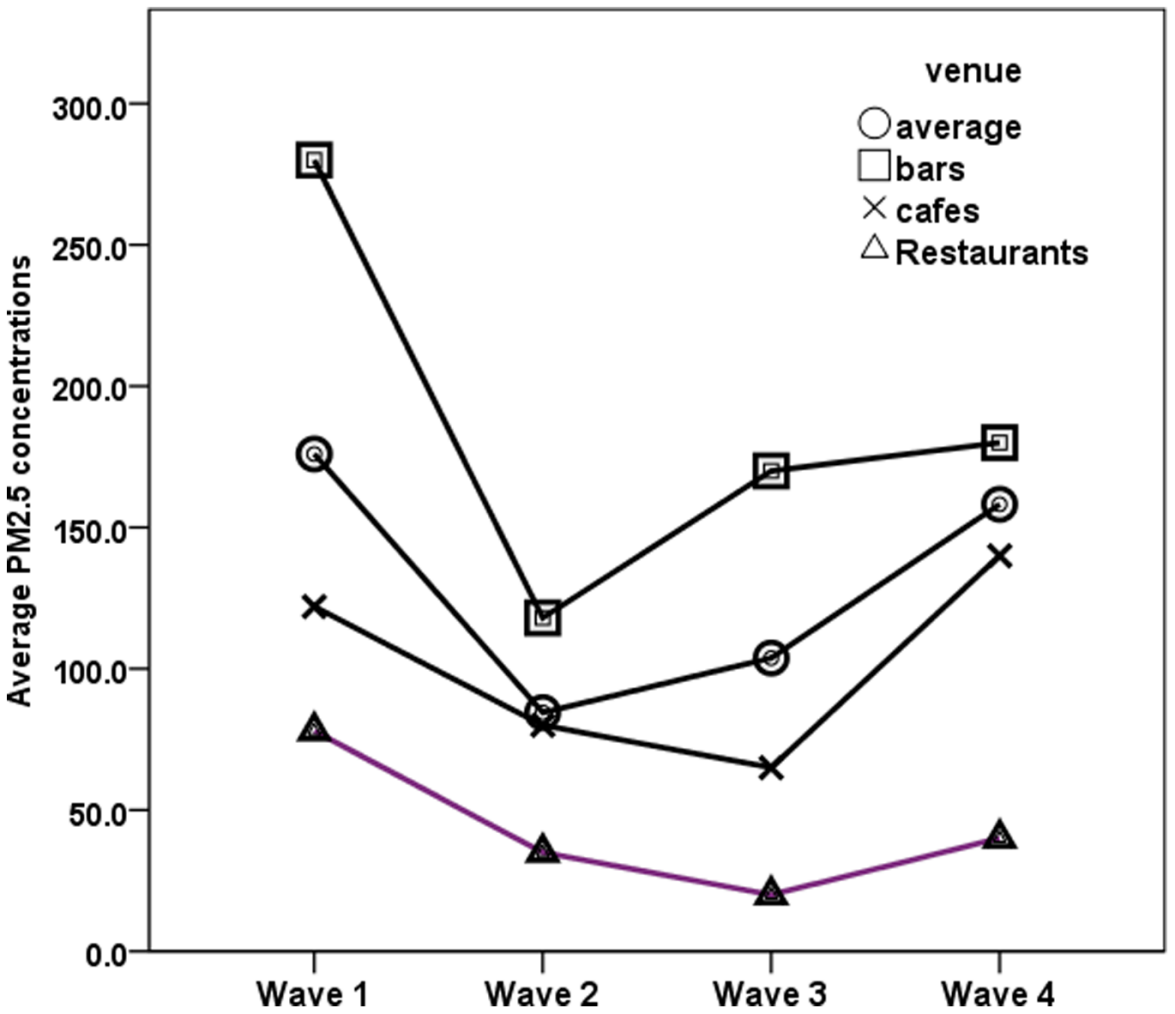
Temporal trends in mean PM_2.5_ concentrations within venues of the Hellenic Air Monitoring Study, Greece, 2010–2011.

Following the implementation of the 2010 smoke-free legislation, mean PM*_2.5_* concentrations attributable to SHS declined significantly from 175.3 µg/m^3^ pre-ban (Wave 1) to 84.52 µg/m^3^ immediately post-ban (Wave 2) (p<0.001) (**Figure 1**). The temporal trend in absolute PM*_2.5_* levels indicated that, following the lower SHS concentrations in Wave 2, mean SHS concentrations were higher in Wave 3 (103.8 µg/m^3^) and Wave 4 (158.2 µg/m^3^). Comparing Wave 2 to Wave 3 measurements, no statistically significant difference was noted (p = 0.225). However, Wave 4 indoor SHS concentrations were statistically higher than in Wave 2 (p<0.001) or Wave 3 (p = 0.026). The difference in absolute PM*_2.5_* concentrations attributable to SHS is portrayed in **Table 2**. However, within the multivariate analysis controlling for ventilation, time of day, day of week, city and venue type, all post-ban measurements were still lower than Wave 1 (pre-ban) measurements (beta: −118.7, for Wave 2, beta: −87.6 for Wave 3, and beta: −69.9 in Wave 4) (**Table** **3**). Moreover, controlling for the above potential confounders, venue type was a very critical predictor of the mean SHS concentrations in bars and cafes, which had higher mean PM*_2.5_* concentrations throughout all measurements, (beta: −91.6 µg/m^3^ and beta: −59.6 µg/m^3^) compared to restaurants (p<0.001). Open windows, doors and walls led to lower indoor PM*_2.5_* concentrations attributable to SHS (beta: −76.7 µg/m^3^, p<0.001), as did measurements taken during the day in comparison to those performed after 8 pm (beta: −27.5 µg/m^3^, p = 0.032).

**Table 2:** Temporal trends in mean indoor PM*_2.5_* measurements (µg/m^3^) and unadjusted pair-wise comparisons between Waves of the Hellenic Air Monitoring Study, Greece 2010–2011.

Wave (n)	Mean PM_2.5_ (95%CI)	St. err	Range min–max	*vs.* Wave 1	*vs.* Wave 2	*vs.* Wave 3	*vs.* Wave 4
**Wave 1 (148)**	175.3 (134.4, 216.3)	20.7	1–2480	–	<0.001	0.024	0.587
**Wave 2 (146)**	84.5 (80.5, 101.4)	7.4	0–373	<0.001	–	0.225	<0.001
**Wave 3 (75)**	103.8 (70.3, 137.3)	16.8	2–772	0.024	0.225	–	0.026
**Wave 4 (76)**	158.2 (123.6, 192.8)	17.4	3–654	0.587	<0.001	0.026	–

**Abbreviations**: St. Error = Standard error; PM_2.5_ = Particulate matter ≤2.5 microns in diameter; n = number of wave-specific sampled sites; 95%CI = 95% Confidence Interval.

**Table 3:** Factors associated with indoor PM_2.5_ exposure attributable to SHS exposure in venues (N = 455) throughout Greece, the Hellenic Air Monitoring Study, 2010–2011.

	Adj. Beta^1^ (µg/m^3^)	St. Error	*p*-value	Crude Beta (µg/m^3^)	St. Error	*p*-value
**Wave**						
* 1 (Partial ban)*	ref	ref.	ref.	ref	ref.	ref.
* 2 (Complete ban)*	−118.70	20.84	<0.001	−82.71	19.66	<0.001
* 3 (Complete ban)*	−87.57	24.09	<0.001	−66.96	24.53	0.007
* 4 (Complete ban)*	−69.80	29.00	0.017	−13.68	27.16	0.615
**Venue type**						
* Restaurant (Ref)*	ref	ref.	ref.	ref	ref.	ref.
* Cafe*	59.57	20.13	0.004	51.72	20.64	0.013
* Bar*	91.60	21.11	<0.001	106.02	20.77	<0.001
**City**						
* Athens (Ref)*	ref	ref	ref	ref	ref.	ref.
* Thessaloniki*	−15.80	20.51	0.442	3.70	20.36	0.856
* erres*	−56.81	23.54	0.017	−68.27	22.26	0.003
* Larissa*	−45.79	22.11	0.040	−47.53	22.47	0.035
* Crete*	−41.46	20.25	0.042	−71.06	20.04	<0.001
**Windows**						
* Closed (Ref)*	ref	ref.	ref.	ref	ref.	ref.
* Open*	−76.74	13.45	<0.001	−50.70	12.30	<0.001
**Time of day**						
* Night (Ref)*	ref	ref.	ref.	ref	ref.	ref.
* Day*	−27.53	12.75	0.032	−37.04	12.39	0.003
**Day of week**						
* Weekend (Ref)*	ref	ref.	ref.	ref	ref.	ref.
* Mon–Thu*	−13.26	11.93	0.268	−5.01	11.91	0.675

**Abbreviations**: Adj. Beta = Adjusted Beta coefficient; St. Error = Standard error; Ref = Reference Category; PM_2.5_ = Particulate matter ≤2.5 microns in diameter; SHS = secondhand smoke; N = total number of sampled sites.

1 Mixed effects Linear regression analyses adjusted for all other variables in the table.

Similar results were derived when smoker density and average number of lit cigarettes per venue were also successfully used as proxies of enforcement, indicating their potential use as indicators of success of smoke-free legislation (**Table 4** **and** **Table 5**).

**Table 4:** Adjusted^1^ and crude linear regression models for the relationship between venue and measurement characteristics and average number of cigarettes^2^ within hospitality venues in Greece (N = 445), 2010–2011.

Variable	Adj. Beta (Sticks)	St. Error	*p*-value	Crude Beta (Sticks)	St. Error	*p*-value
**Wave**						
* 1 (Partial ban)*	ref	ref.	ref.	ref	ref.	ref.
* 2 (Complete ban)*	−1.76	0.40	<.0001	−0.56	0.14	<.001
* 3 (Complete ban)*	−0.80	0.56	0.158	−0.49	0.19	0.011
* 4 (Complete ban)*	−0.90	0.59	0.133	−0.61	0.17	0.000
**Venue type**						
* Restaurant (Ref)*	ref	ref.	ref.	ref	ref.	ref.
* Cafe*	2.39	0.76	0.002	0.59	0.31	0.057
* Bar*	3.92	0.78	<.001	1.40	0.30	<.001
**City**						
* Athens (Ref)*	ref	ref.	ref.	ref	ref.	ref.
* Thessaloniki*	−0.21	0.78	0.788	−0.71	0.29	0.017
* Serres*	0.05	0.82	0.953	−0.86	0.31	0.007
* Larissa*	−1.07	0.82	0.193	−0.22	0.33	0.510
* Crete*	0.05	0.77	0.948	−1.13	0.30	<.001
**Windows**						
* Closed (Ref)*	ref	ref.	ref.	ref	ref.	ref.
* Open*	−0.63	0.36	0.086	0.18	0.13	0.151
**Time of day**						
* Night (Ref)*	ref	ref.	ref.	ref	ref.	ref.
* Day*	−1.10	0.38	0.004	−0.44	0.14	0.002
**Day of week**						
* Weekend (Ref)*	ref	ref.	ref.	ref	ref.	ref.
* Mon–Thu*	−0.39	0.34	0.253	0.12	0.13	0.327

**Abbreviations**: Adj. Beta = Adjusted Beta coefficient; St. Error = Standard error; Ref = Reference Category; N = total number of sampled sites; BC/100 m^3^ = Burning cigarettes per 100 m^3.^

1 Adjusted for all other variables in the table.

2 Average number of cigarettes per measurement.

**Table 5:** Adjusted^1^ and crude linear regression models for the relationship between venue and measurement characteristics and smoker density^2^ levels, within hospitality venues in Greece (N = 445), 2010–2011.

Variable	Adj. Beta (BC/100 m^3^)	St. Error	*p*-value	Crude Beta (BC/100 m^3^)	St. Error	*p*-value
**Wave**						
* 1 (Partial ban)*	ref	ref.	ref.	ref	ref.	ref.
* 2 (Complete ban)*	−0.54	0.17	0.001	−0.56	0.14	<.001
* 3 (Complete ban)*	−0.53	0.20	0.008	−0.49	0.19	0.011
* 4 (Complete ban)*	−0.55	0.19	0.006	−0.61	0.17	<.001
**Venue type**						
* Restaurant (Ref)*	ref	ref.	ref.	ref	ref.	ref.
* Cafe*	0.57	0.31	0.071	0.59	0.31	0.057
* Bar*	1.11	0.32	0.001	1.40	0.30	<.001
**City**						
* Athens (Ref)*	ref	ref.	ref.	ref	ref.	ref.
* Thessaloniki*	−0.53	0.30	0.081	−0.71	0.29	0.017
* Serres*	−0.48	0.34	0.159	−0.86	0.31	0.007
* Larissa*	−0.19	0.34	0.580	−0.22	0.33	0.510
* Crete*	−0.87	0.32	0.007	−1.13	0.30	<.001
**Windows**						
* Closed (Ref)*	ref	ref.	ref.	ref	ref.	ref.
* Open*	−0.04	0.14	0.770	0.18	0.13	0.151
**Time of day**						
* Night (Ref)*	ref	ref.	ref.	ref	ref.	ref.
* Day*	−0.33	0.15	0.024	–0.44	0.14	0.002
**Day of week**						
* Weekend (Ref)*	ref	ref.	ref.	ref	ref.	ref.
* Mon–Thu*	0.004	0.14	0.979	0.12	0.13	0.327

**Abbreviations**: Adj. Beta = Adjusted Beta coefficient; St. Error = Standard error; Ref = Reference Category; N = total number of sampled sites; BC/100 m^3^ = Burning cigarettes per 100 m^3^.

1 Adjusted for all other variables in the table.

2 Average number of cigarettes/100 m^3^ of venue air volume.

### Signage and Ashtrays as Indicators of Enforcement

During Wave 4, outdoor signs banning smoking within the premises were present in only about a fifth of the venues (20.8%), while 8.3% had pro-smoking signs. Overall, 60% of venues had an indoor sign banning smoking, of which the vast majority (90.7%) had signs on the interior walls banning smoking, while only less than one-fifth (18.6%) had signs on table-tops or bar-tops as mandated by the existing regulation. As seen in **Table 6**, after adjusting for city and venue type in a longitudinal analysis of Waves 3 and 4, the existence of outdoor signage banning smoking was not found to affect indoor PM_2.5_ concentrations (beta difference of −10.9, p = 0.667), with mean PM_2.5_ concentrations of 167 µg/m^3^ vs. 159 µg/m^3^ (absence/presence of outdoor antismoking signage respectively). Indoor signage was also not found to reduce indoor SHS concentrations, even after controlling for other factors (beta −18.1, p = 0.464) (mean PM_2.5_: 172 µg/m^3^ vs. 159 µg/m^3^; absence/presence of indoor antismoking signage respectively),

**Table 6:** The relationship between ashtrays and signage within venues (n = 151) and adherence to a smoke-free legislation, within Waves 3 and 4 of the Hellenic Air Monitoring Study Greece, 2010–2011.

Enforcement determinants	Adj. Beta^1^ (µg/m^3^)	St. Error	*p*-value	Crude Beta (µg/m^3^)	St. Error	*p*-value
**Ashtray**						
* Ashtrays or equivalents* 2	67.11	28.06	0.017	122.43	25.11	<0.001
**Anti-smoking signage**						
* Any signage against smoking* 3	−10.89	25.32	0.667	−9.22	26.50	0.728
* Indoor signage against smoking*	−18.16	24.83	0.464	−19.90	26.18	0.447
* Outdoor signage against smoking*	−15.68	27.43	0.568	17.00	30.79	0.581
**Pro-smoking signage**						
* Signage against banning indoor smoking*	1.41	37.05	0.970	52.4	38.6	0.175

**Abbreviations**: Adj. Beta = Adjusted Beta coefficient; St. Error = Standard error; n = number of sampled sites.

1 Adjusted for venue type and city in a mixed effects linear model accounting for repeated measures in Waves 3 and 4.

2 Includes both factory made receptacles as well as improvised ashtray equivalents.

3 Refers to presence of any signage either outdoors or indoors against smoking (signs on doors, walls, bar tops and table tops against smoking).

In contrast to signage, ashtray or ashtray equivalents were strong determinants of indoor SHS concentrations and legislation breaches. In the most recent measurement (Wave 4), 59.7% of the hospitality venues in Greece had ashtrays available, while 33.3% had some form of ashtray equivalent, such as a candleholder and cups. The presence of ashtrays was strongly associated with city (p = 0.001) and with venue type (p = 0.036), while the mean PM_2.5_ in venues with ashtrays was 196.85 µg/m3 vs. 57.02 µg/m3 in sites with no ashtrays (P<0.001). Adjusting for city and venue type, mean PM_2.5_ levels were higher in hospitality sites with ashtray or ashtray equivalents compared to those without (beta: +67 µg/m^3^, p = 0.017), indicating the potential role of the ashtray as an optical smoking cue and indicator of establishment noncompliance with the smoke-free legislation.

## Discussion

Our results indicated that, immediately following the implementation of the 2010 smoking ban in Greece, SHS concentrations dropped by 50%. However, concentrations subsequently increased over following Waves to slightly lower than pre-ban concentrations. This finding indicates that population support may not be sufficient to sustain smoke-free legislation if enforcement is absent, as corroborated by the lack of enforcement noted throughout Greece. Additionally, a very novel finding was that two markers of enforcement, i.e. signage and ashtrays, were assessed within this study. The latter strongly related to indoor SHS concentrations, even after controlling for other factors.

Extensive research has elucidated the social and demographic characteristics of those supportive of smoke-free environments [11]. Indeed, non-smoking, more affluent and higher educated individuals are more likely to proactively support smoking bans in public places and engage a potential violator [12]. Population support of smoke-free laws is known to increase with increased duration from onset of implementation as the benefits of smoke-free legislation become more evident. [13]. However, it is possible that population support may not be sufficient to maintain smoke-free legislation, especially if enforcement is inadequate. In addition, a lack of enforcement often leads to an “avalanche effect”. This occurs when one non-compliant venue leads to another until compliance is minimal and the indoor concentrations of SHS exposure slowly revert to the previous, pre-legislation levels. While some countries such as Scotland (246 µg/m^3^ pre- to 20 µg/m^3^ post ban) and Italy (119 µg/m^3^ to 38 µg/m^3^) have noted almost a complete elimination of indoor SHS exposure, others such as Norway (262 µg/m^3^ to 77 µg/m^3^) and Israel (245 µg/m^3^ to 161 µg/m^3^) have noted reductions similar to those following the implementation of the legislation in Greece [14]–[17].

Hospitality venues may strongly influence whether people smoke or not as “social smoking” is very common, especially among youth [18]. Among smokers, visual smoking stimuli presented in the natural environment may stimulate robust craving effects, thus acting as a strong cue for those who would have refrained from smoking or former smokers who wish to remain abstinent [19]. Indeed, research among both adults and youth has indicated that social situations comprise the majority of addictive relapse situations [20], [21]. It is likely that a smoker without cues of social acceptability of smoking would refrain from lighting a cigarette in a non-smoking venue, and could be proactively confronted by non-smokers hoping to retain a smoke-free environment [12]. While factors such as venue type, day of week, time of day, city and ventilation characteristics have been identified as determinants of indoor SHS concentrations, one of the innovative findings of this study was the fact that ashtrays or ashtray equivalents within a venue was a strong proxy for indoor air SHS concentrations and legislation enforcement. The existence of ashtrays, which our study indicates was associated with higher levels of indoor SHS exposure, may be perceived as a sensory cue and proxy for the social acceptability of smoking within the venue and management’s consent to smoking within the establishment.

Signage has been previously indicated to play a potential role in self-enforcement, acting as a reminder of the smoke-free policy. These are crucially important factors in achieving employee and community awareness [22], [23]. While signage may be important initially, as patrons and employees become more aware that smoking is not permitted in the venue, its role may diminish over time, as indicated by the reduction in number of signs noted at the 5-year follow up of the successful Scottish ban [24]. However, within our analysis, signage was not found to be a strong determinant of indoor SHS concentrations. This is most likely due to the fact that its positive reinforcing role was dwarfed by the negative influence of ashtrays.

A factor that could have partially contributed to the noted reduction in SHS concentrations is the reduction in smoking prevalence noted over the past few years within Greece. Notably, through the combination of tax increases, educational campaigns, advertising restrictions and the implementation of smoke-free legislations, between 2006 and 2010, current smoking prevalence among young adults dropped from 48% to 35%, a factor which could have aided the reduction in SHS concentrations [25]. However, we must state that the adoption and maintenance of smoke-free legislation is dictated not only by the society, but also by political will and enforcement agencies. Subsequently, we must note that between Wave 2 and Wave 3, national elections took place in Greece, causing cabinet reshuffling after Wave 2 and the second minister oscillating between enforcement and repeal of the law [26]–[28]. This change in political leadership may have also played an important role in reducing the effectiveness of the smoke-free legislation during Waves 3 and 4, suggesting that strong political leadership may be critically-important in enforcing smoke-free legislation. While political will was lacking during Waves 2 and 3, it is interesting to note that population support for the smoke-free legislation remained substantially high, as 75% of the population had indicated that they were supportive of the legislation, while over 16,000 telephone complaints were made over the first 6 months of implementation [29].

The novelty of this study is supported by the unique environment, allowing us to assess the evolution of non-enforced smoke-free legislation. The large number of venues used (n = 150 at baseline, 75 at Wave 4) and the multi-venue measurements (n = 455) make this air monitoring study one of the largest ever performed at a national level. However, while indoor air measurements were performed prospectively, the findings regarding signage and the existence of ashtrays were derived from a pooled analysis of Wave 3 and Wave 4, as these factors were not measured in Waves 1 and 2. However, this approach allowed us to control for other factors influencing indoor SHS concentration such as day, time, ventilation and venue type. While our results are novel, further research is needed among other populations to expand the generalizability of these findings to other countries. Within this analysis we did not proceed to assess the potential factors that could be related to the adherence or not to the smoke-free legislation, which could include the social and environmental changes noticed within Greece, or the reduction in smoking prevalence and consumption in general [25]. Further research is needed to elucidate how these factors might have influenced indoor SHS concentrations also.

In conclusion, other than indicating the success and pitfalls of the Greek national smoking ban, our findings have several novel implications for countries contemplating banning smoking in the hospitality industry. Firstly, within the initial phase of implementation, while public support is sufficient to maintain smoke-free legislation, compliance may decline rapidly if enforcement is limited or non-existent. Secondly, the existence of ashtrays or ashtray equivalents within a venue is a strong determinant of indoor SHS exposure, indicating that comprehensive enforcement should include complete removal of ashtrays and other related objects acting as cues for smoking within a venue, thereby helping to denormalize tobacco use.
